# Therapeutic Anticoagulation Impacts MR Morphologic Recurrence Patterns in Glioblastoma—A Matched-Pair Analysis

**DOI:** 10.3390/jcm11020422

**Published:** 2022-01-14

**Authors:** Daniel Dubinski, Sae-Yeon Won, Bedjan Behmanesh, Max Dosch, Viktoria Puchinin, Peter Baumgarten, Joshua D. Bernstock, Martin Voss, Patrick Schuss, Jürgen Konczalla, Marcus Czabanka, Thomas M. Freiman, Florian Gessler

**Affiliations:** 1 Department of Neurosurgery, University Hospital, Goethe University, 60528 Frankfurt, Germany; Sae-Yeon.Won@med.uni-rostock.de (S.-Y.W.); bedjan.behmanesh@gmail.com (B.B.); maxpdosch@gmail.com (M.D.); vicky.puchinin@gmail.com (V.P.); peter.baumgarten@med.uni-jena.de (P.B.); Juergen.Konczalla@kgu.de (J.K.); Marcus.Czabanka@kgu.de (M.C.); 2 Department of Neurosurgery, University Medicine Rostock, 18055 Rostock, Germany; Thomas.Freiman@med.uni-rostock.de (T.M.F.); Florian.Gessler@med.uni-rostock.de (F.G.); 3 Department of Neurosurgery, Birgham and Women’s, Harvard Medical School, Boston, MA 02115, USA; JBERNSTOCK@bwh.harvard.edu; 4 Dr. Senckenberg Institute of Neurooncology, Goethe University Hospital, 18055 Frankfurt, Germany; Martin.Voss@kgu.de; 5 Department of Neurosurgery, Unfallkrankenhaus Berlin, 12683 Berlin, Germany; patrick.schuss@ukb.de

**Keywords:** pulmonary embolism, therapeutic anticoagulation, recurrence pattern, glioblastoma survival

## Abstract

Background: Glioblastoma (GBM) patients are at particularly high risk for thrombotic complications. In the event of a postoperative pulmonary embolism, therapeutic anticoagulation (tAC) is indispensable. The impact of therapeutic anticoagulation on recurrence pattern in GBM is currently unknown. Methods: We conducted a matched-pair cohort analysis of 57 GBM patients with or without tAC that were matched for age, sex, gross total resection and MGMT methylation status in a ratio of 1:2. Patients’ characteristics and clinical course were evaluated using medical charts. MRI characteristics were evaluated by two independent authors blinded to the AC status. Results: The morphologic MRI appearance in first GBM recurrence showed a significantly higher presence of multifocal, midline crossing and sharp demarcated GBM recurrence patterns in patients with therapeutic tAC compared to the matched control group. Although statistically non-significant, the therapeutic tAC cohort showed increased survival. Conclusion: Therapeutic anticoagulation induced significant morphologic changes in GBM recurrences. The underlying pathophysiology is discussed in this article but remains to be further elucidated.

## 1. Introduction

Glioblastomas (GBMs) are one of the most aggressive cancer entities in oncology, with dismal prognosis despite multimodal therapy. GBM patients suffer from a hypercoagulable condition that varies from a subclinical asymptomatic hypercoagulable state to manifest thrombosis of the large vessels. On the clinical level, the cumulative probability of thrombotic complications in GBM patients ranges between 20% and 30% per year of survival and remains higher than other malignancies throughout the course of the disease [1]. On the subclinical level, the hypercoagulable state contributes to the appearance of micro-thrombosis and consecutive necrosis, which is in turn associated with tumor malignancy and poor prognosis [2]. GBM cells directly induce the hypercoagulable condition by overproducing tissue factor (TF), the main activator of coagulation, which promotes thrombin generation and the activation of fibrin deposition [3,4]. Circulating TF is elevated in GBM patients, and TF levels are directly correlated with D-dimer levels [5,6,7]. On the molecular level, elevated TF and consecutive thrombin activation lead to several cancer-progression mechanisms via the protease-activated receptor (PAR) proteins, such as the overexpression of angiogenesis-related proteins like TF, VEGF, VEGF-R, and metalloproteinase 2 (MMP-2) [5,8,9].

However, once GBM patients experience a thromboembolic event, tAC is indispensable. In principle, direct oral anticoagulants (DOACs) or low-molecular-weight heparins (LMWHs) are available for this purpose despite the most often present contraindication, and are thus used as off-label medications [10]. We recently reported our analysis of a comparison between both agents for tAC in GBM patients with VTE, where GBM patients with DOACs showed increased survival compared to those on LMWHs [11].

In this study, we hypothesized that GBM patients with tAC would show morphologic MR-detectable changes in tumor recurrence. According to our hypothesis, factor Xa inhibition through LMWHs or DOACs could counteract the TF-mediated overexpression of hypoxia-related proteins and lead to detectable MR-morphological changes in GBM relapse.

## 2. Materials and Methods

### 2.1. Patients and Data Collection

For this retrospective matched-pair analysis, ethical approval was obtained from the Ethics Committee of the University Hospital Frankfurt, Germany (Identification number: 20-683) and the Ethics Committee of the University Medicine Rostock, Germany (Identification number: A 2021-0112). As a non-interventional bi-centric study, no patient consent was necessary. All patients who underwent craniotomy for tumor resection and had the radiologically confirmed diagnosis of PE from 2012 to 2019 were included. Treatment decisions, including determination for surgery, were rendered by the local interdisciplinary tumor board. Patient follow-up was achieved in the outpatient neurosurgical department. Included data on patient characteristics and clinical course were evaluated through the chart record. Exclusion criteria were the lack of cranial MRI, thoracic CT scan or incomplete follow-up chart.

### 2.2. Image Analysis

**Figure 1:**
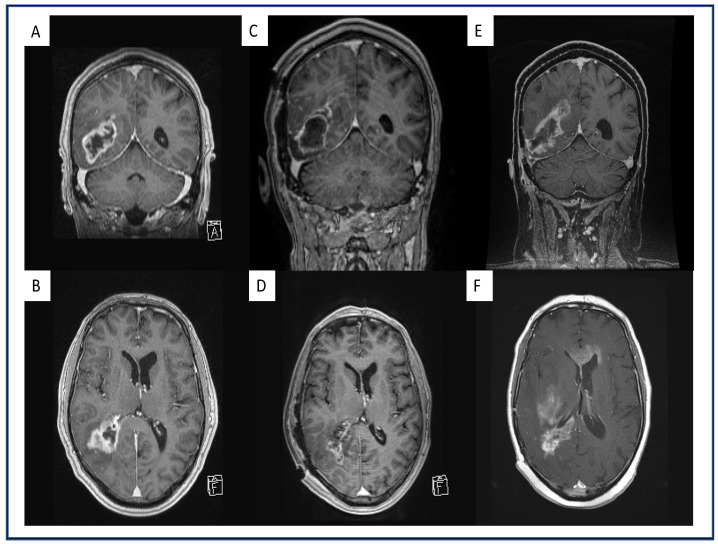
Exemplary representation of a multifocal, midline-crossing GBM under therapeutic AC on the first recurrence after standard-of-care treatment. Preoperative coronal (**A**) and axial (**B**) gadolinium-enhanced, T1-weighted MRI of a GBM patient and a right parieto-occipital contrast-enhancing lesion. Postoperative coronal (**C**) and axial (**D**) gadolinium-enhanced, T1-weighted MRI of the same patient after GTR where no contrast enhancement is present. Coronal (**E**) and axial (**F**) gadolinium-enhanced, T1-weighted MRI of the same patient with therapeutic AC upon first progression with multifocal, midline-crossing GBM recurrence in the right hemisphere.

**Figure 2:**
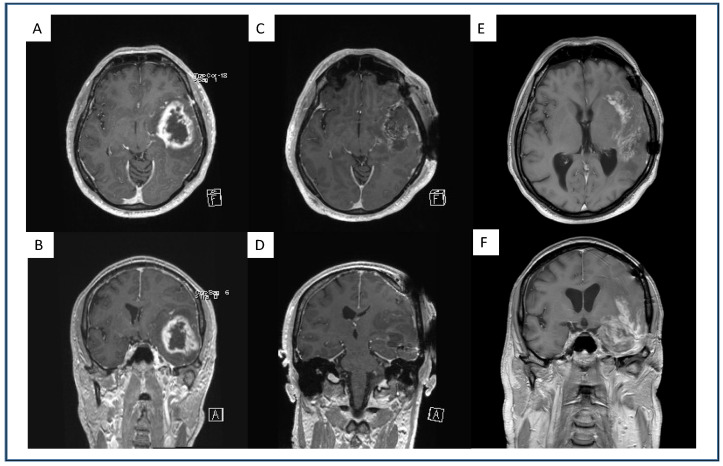
Exemplary representation of a dull demarcated GBM under therapeutic AC, on the first recurrence after standard-of-care treatment. Preoperative axial (**A**) and coronal (**B**) gadolinium-enhanced, T1-weighted MRI of a GBM patient with a left fronto-temporal contrast-enhancing lesion. Postoperative axial (**C**) and coronal (**D**) gadolinium-enhanced, T1-weighted MRI of the same patient after GTR where no contrast enhancement is present. Axial (**E**) and coronal (**F**) gadolinium-enhanced, T1-weighted MRI of the same patient with therapeutic AC upon first progression with a dull demarcated recurrent lesion.

Image analysis was performed by two neurosurgeons (B.B. and S.Y.-W.) that were blinded to patients’ AC status. Preoperative, postoperative and first progression tumors were analyzed with IPlannet 3 (Cranial planning software, Brainlab AG, Feldkirchen, Germany). A representative analysis is displayed in Figure 1 and Figure 2. Tumor was defined as contrast-enhanced T1-weighted lesions. Recurrence patterns were defined as local, distant, multifocal or diffuse according to the radiographic classification as described by Pope [12].

Local recurrence (contrast enhancing or not) was defined as unifocal, contiguous with the primary site or resection cavity. Multifocal recurrence was defined as three or more non-contiguous lesions (contrast enhancing or not) including the primary site. Furthermore, the following characteristics were analyzed: midline crossing vs. intrahemispheric lesion, ring enhancing vs. inhomogeneous lesion and intratumoral hemorrhage vs. no intratumoral hemorrhage as well as sharp vs. dull tumor demarcation. The postoperative extent of resection was calculated by the pre- and postoperative tumor volume. Gross total resection (GTR) was defined as complete removal (100%) of contrast-enhancing tissue.

### 2.3. Patient Follow-Up

The postoperative period was defined as the time from operation to discharge from neurosurgery. After discharge, patient follow-up was carried out in the department of neuro-oncology every 3 months until the transfer to palliative care or hospice. The response assessment in neuro-oncology (RANO) criteria were conducted by an attending neuro-radiologist. In detail, cranial MRI including standard sequences (T1-weighted (w), T2-w, T2*-w, FLAIR, diffusion-weighted imaging (DWI), T1-w with contrast agent) and a T1-w sequence with contrast agent was performed.

### 2.4. Statistics

Data analysis was performed with IBM SPSS Statistics Version 23.0 (SPSS Inc., IBM Corp., Armonk, NY, USA). For patient and tumor MRI characteristics, descriptive statistics were used. Fisher’s exact test was used for the comparison of categorical variables between the cohorts. For continuous parameters, the Wilcoxon/Mann–Whitney test was used. To assess the impact of the variables, odds ratios (ORs) with 95% confidence intervals (CIs) were calculated. Results with *p* ≤ 0.05 were considered statistically relevant. To estimate the survival rates, Kaplan–Meier analysis was used. The differences between curves were assessed using the log-rank test. Overall survival (OS) was defined as the time of first presentation to death.

## 3. Results

In total 57 patients were included in the final analysis. We matched patients for age, sex, GTR and MGMT methylation status in a ratio of 1:2. Thus, 19 patients received therapeutic AC for PE vs a matched cohort of 38 GBM patients without therapeutic AC (Table 1).

The distributions of AC in the therapeutic AC cohort were as follows: rivaroxaban (Xarelto^®^ Bayer AG, Leverkusen, North Rhine-Westphalia, Germany) *n* = 6, edoxaban (Lixiana^®^ Daiichi Sankyo Europe GmbH, Pfaffenhofen Bavaria, Germany) *n* = 8 and LMWH (Clexane^®^ Sanofi-Aventis Deutschland GmbH, Frankfurt am Main, Hesse, Germany) *n* = 5. However, during the follow up no additional cardiovascular events were recorded for both cohorts. Univariate analysis revealed that patients on therapeutic AC had a significantly higher rate of multifocal recurrence (47% vs. 16%), whereas patients without tAC more often showed local recurrence (53% vs. 84%), (*p* = 0.02). Furthermore, patients on tAC showed a significantly higher percentage of midline-crossing recurrence (47%) compared to patients without therapeutic AC (11%; *p* = 0.004). A patient example with a multifocal, midline-crossing recurrence is displayed in Figure 1.

We observed no significant difference in the presence of intratumoral hemorrhage between the groups. However, patients on tAC showed a significantly higher percentage of sharp demarcated recurrence (37%) compared to patients without therapeutic AC (11%; *p* = 0.03). A patient example with a dull demarcated recurrence is displayed in Figure 2.

**Figure 3:**
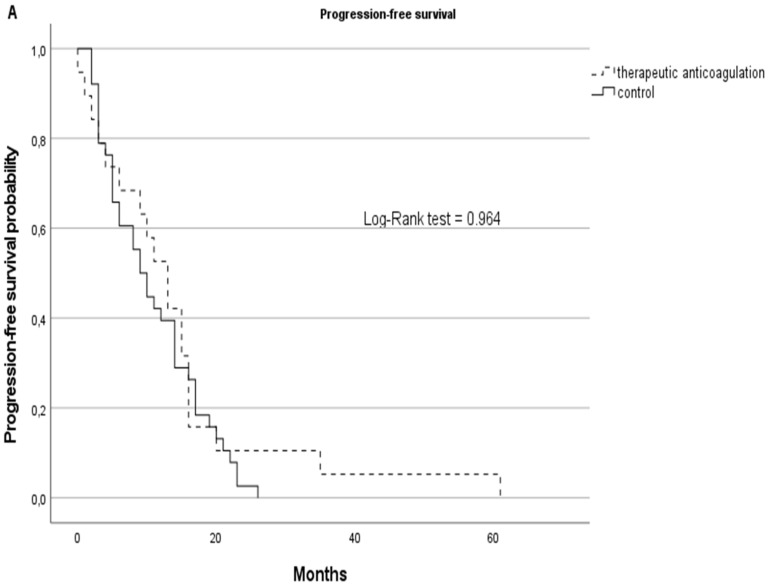

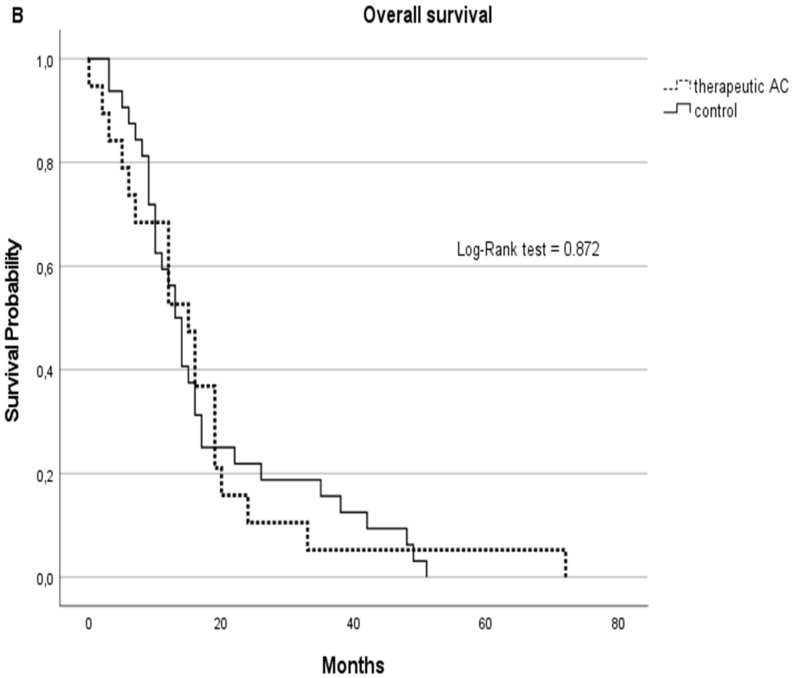
PFS (**A**) and OS (**B**) represented by Kaplan–Meier curves for GBM patients on therapeutic anticoagulation (tAC) represented with dashed lines vs. the GBM control cohort without tAC, represented with solid lines.

Although statistically non-significant, the therapeutic AC cohort showed better survival rates with a median of 13 months in PFS (SD: 14) vs. 9.5 (SD:7) months in the non-AC group and a median OS of 15 months (SD: 15) vs. 12.5 (SD: 13) months in the non-AC group. Kaplan–Meier curves are displayed in Figure 3.

## 4. Discussion

Our study demonstrates a significant difference in the MRI-morphologic appearance of the first recurrence in GBM patients with therapeutic anticoagulation compared to a matched cohort without tAC. GBM patients on tAC had a significantly higher rate of multifocal, midline-crossing and sharply demarcated lesions.

### 4.1. Multifocal and Midline-Crossing Relapse

Several studies have investigated the role of MRI-morphologic appearance in GBM recurrence. Pope et al. distinguished between local, distant, diffuse and multifocal GBM recurrence [12]. In his study, most GBM relapses were of local character, and in that form associated with better prognosis compared to the other radiographic characteristics at the time of progression. Several preceding studies confirmed the local relapse form as predominant, and reported numbers of up to 90% [13,14,15,16]. While the control group in our analysis was in line with the reported data with a predominant local recurrence, patients on tAC showed a significant association with multifocal and midline-crossing recurrence patterns. This observation is challenging. When discussing the carcinogenicity of AC, the literature regarding an interaction between AC and tumor cells is inconclusive. While the majority of clinical studies describe a survival benefit for cancer patients on AC, experimental animal models have shown pro-neoplastic effects where AC was shown to induce tumor growth mainly via trypsin inhibition [17,18,19]. This observation could hint at an AC-induced pro-neoplastic effect with increased tissue invasion. However, while this hypothesis could explain the MRI-detected overrepresentation of multifocal, midline-crossing GBM recurrence in tAC patients, the molecular evidence needs to be investigated further.

### 4.2. Sharp Demarcated Lesions

The discovery of significant overrepresented, sharply demarcated (hence less infiltrative) GBM relapse in our tAC cohort is exciting. Most of the scientific literature agrees on the fact that low tumor oxygenation promotes tumor invasion into the healthy brain parenchyma, supposedly in order to evade the adverse microenvironment [9,20,21]. Therapeutic anticoagulation, on the other hand, could counteract this molecular mechanism by reducing the rate of intratumoral micro-thrombosis and could, at least in part, explain the significant overrepresentation of sharply demarcated relapse in our tAC cohort as an indicator of reduced tissue hypoxia and decreased parenchyma invasion. Another explanatory approach for the sharp demarcation finding could lie in the complex interplay between anticoagulants and cell adhesion proteins. Several studies have shown that anticoagulation therapy is associated with positive outcome in solid cancer entities, with mechanisms beyond their classical anticoagulatory effect. Platelets, for example, were shown to act as a bridge connecting cancer cells to the endothelial layer, thereby enhancing cancer cell attachment, tumor progression and escape from immunologic surveillance. AC drugs were shown to repeal this association via the downregulation of several adhesion proteins, including cadherins and selectins [22,23]. While the AC-related anti-cancer properties should be subjected to further investigation, our finding could add important clinical reference for future studies.

### 4.3. Analysis of Intratumoral Hemorrhage

Intratumoral hemorrhage was not observed in patients on tAC upon first recurrence. Preceding studies indicated a hemorrhage risk of DOACs and LMWHs in the treatment of cancer-associated venous thromboembolism [24,25]. However, recent studies were able to show a satisfactory safety profile in DOACs for PE treatment in GBM [11,24]. Our finding is therefore in line with the current literature on DOACs in GBM.

### 4.4. Progression-Free and Overall Survival

Thromboembolic complications contribute to patient morbidity and mortality and constitute a well-established risk factor for poor outcome in GBM. However, in our cohort, patients with PE and consecutive tAC showed no significant difference in PFS or OS. In fact, patients receiving tAC even showed a tendency of increased survival. Since data on GBM patients with tAC are rare, the explanatory approach can be only hypothetical. Several studies of extracranial malignancies described significant survival benefits in patients on anticoagulation regimes. Possible explanations include the interference of AC with cancer-cell proliferation, tumor growth, angiogenesis, immune system evasion and drug resistance [26,27]. However, in a retrospective analysis Le Rhun et al. found that AC use was not associated with improved OS in GBM patients [28].

In our cohort, on the contrary, the AC-related anti-cancer properties presumably outweighed the negative side effects of PE in GBM relapse. It is therefore possible that the association between thrombosis and cancer masks potential AC-mediated survival benefits.

### 4.5. Limitations

The obvious limitation of this clinical study is the small sample size and the retrospective design. As this part is of observational character, confounding, selection bias, reverse causation and uncontrolled statistical error risk cannot be excluded. Due to the study design, we cannot exclude the possibility of a selection bias in our cohort. The manifestation of PE itself could indicate an altered TF concentration and/or unknown tumor characteristics that led to the hypercoagulable state in these patients. This issue should be addressed in future prospective studies. However, further prospective randomized trials with large cohorts are necessary to validate our findings. As a strength, our investigation is the first study to investigate the question of tAC effects on tumor progression in GBM.

### 4.6. Summary

The clinical analysis presented here can only point to a possible link between therapeutic anticoagulation and tumor recurrence. The actual molecular effect of tAC on survival in patients who are already receiving powerful cytotoxic treatment remains to be determined.

## 5. Conclusions

Therapeutic anticoagulation in GBM patients induces a substantial morphological change in the first MRI-detected tumor progression compared to a matched cohort without anticoagulation. The underlying pathophysiology remains speculative, and requires further investigation.

## Figures and Tables

**Table 1:** Uni- and multivariate analysis of juxtaposed characteristics according to therapeutic AC.

Variable	Therapeutic Anticoagulation		Univariate	Multivariate
	Yes (*n* = 19)	No (*n* = 38)	*p*-Value	
Characteristics				
Male, n (%)	9 (47%)	26 (68)	n.s.	n.s.
Age, n (SD)	62 (6)	64 (8)	n.s.	n.s.
Clinical course				
Gross total resection, n (%)	14 (73)	25 (72)	n.s.	n.s.
Subtotal resection, n (%)	5 (27)	13 (28)	n.s.	n.s.
Progression-free survival, months (SD)	13 (14)	9.5 (7)	n.s.	n.s.
Overall survival, months (SD)	15 (15)	12.5 (13)	n.s.	n.s.
Histopathology				
MGMT methylated	10 (53)	15 (40)	n.s.	n.s.
MRI charateristics of reccurent tumor				
Local	10 (53)	32 (84)	0.02	n.s.
Multifocal	9 (47)	6 (16)	0.02	n.s.
Midline crossing	9 (47)	4 (11)	0.004	0.006
No midline crossing	10 (53)	34 (89)	0.004	0.006
Hemorrhage	0 (0)	1 (3)	n.s.	n.s.
No hemorrhage	19 (100)	37 (97)	n.s.	n.s.
Sharp demarcation	7 (37)	4 (11)	0.03	0.008
Without sharp demarcation	12 (63)	34 (89)	0.03	0.008

Abbreviations: MRI: Magnetic resonance imaging; SD: standard deviation; MGMT: O(6)-methylguanine-DNA methyltransferase.

## Data Availability

The data presented in this study are available on request from the corresponding author.
